# Acceptance of COVID-19 Vaccines among Adults in Lilongwe, Malawi: A Cross-Sectional Study Based on the Health Belief Model

**DOI:** 10.3390/vaccines10050760

**Published:** 2022-05-11

**Authors:** Qun Ao, Robert Okia Egolet, Hui Yin, Fuqiang Cui

**Affiliations:** 1 Department of Global Health, School of Public Health, Peking University, Beijing 100191, China; aoqun2018@gmail.com (Q.A.); cuifuq@bjmu.edu.cn (F.C.); 2 Global Health Collaborating Centre for Research and Training in Health Sciences, Peking University, P.O. Box 166, Lilongwe 265, Malawi; egoletroberts@gmail.com; 3 Institute for Global Health and Development, Peking University, Beijing 100191, China

**Keywords:** vaccine acceptance, COVID-19 vaccine, health beliefs model, intention, behavior

## Abstract

The COVID-19 pandemic has had a significant economic and social impact on Malawi. Promoting vaccination is a key protection measure against COVID-19. Employing the health beliefs model (HBM), this study explores various factors that influence COVID-19 vaccination acceptance (intentions and behavior) among adult residents of Malawi. A semi-structured questionnaire was used for data collection. A field-based survey was conducted among adult residents in Lilongwe, Malawi. Descriptive statistics, linear regression, the Chi-square test, and Pearson’s correlation statistics were used for data analysis. A total of 758 questionnaires were involved. Respondents aged 18–24 (OR = 5.079, 95% CI 2.303–11.202), 25–34 (OR = 2.723, 95% CI 1.363–5.438), urban residents (OR = 1.915, 95% CI 1.151–3.187), graduates/professionals (OR = 1.193, 95% CI 0.857–1.651), health workers (OR = 4.080, 95% CI 1.387–12.000), perceived susceptibility (OR = 1.787, 95% CI 1.226–2.605), perceived benefit (OR = 2.992, 95% CI 1.851–4.834), and action cues (OR = 2.001, 95% CI 1.285–3.115) were predictors for “acceptance of COVID-19 vaccine”. The health belief model structure can be used as a good predictor of vaccine acceptance, especially “perceived susceptibility,” “perceived benefit,” and “action cues”. Strengthening COVID-19 vaccine education in these areas will be an important future intervention.

## 1. Introduction

The COVID-19 pandemic has triggered an unprecedented and rapid global public health crisis. As one of the most pressing global threats, the pandemic has affected all aspects of life around the world. Countries have implemented strict precautions and controls to contain the outbreak of COVID-19, such as travel bans and lockdowns [1]. However, new variants, such as Delta and Omicron, are making it harder to contain the epidemic. The development and deployment of vaccines is recognized as one of the most promising health intervention strategies and an important new tool in the fight against COVID-19 [2]. Adequate vaccination coverage can help to reduce infection rates and subsequent mortality from COVID-19. To achieve the goal of containing COVID-19 and returning to normal life, countries need to vaccinate at least 70% of the population in order to build herd immunity against COVID-19. Malawi is a low-income country where public health services are challenged [3]. Controlling the COVID-19 pandemic and conducting vaccination campaigns remain huge challenges for Malawi.

As of 4 December 2021, 1,501,147 vaccine doses have been administered in Malawi. 878,471 and 340,249 people have received the first and second doses of the AstraZeneca vaccine, respectively, while 282,427 people have received a full dose of the Johnson & Johnson vaccine, bringing the total number of fully vaccinated people to 622,676. Malawi currently plans to vaccinate 10.97 million people (60% of the population).

Apart from the scarcity and logistical issues of candidate vaccines, vaccination hesitancy is one of the most critical barriers to achieving mass COVID-19 vaccination rates. According to the World Health Organization, vaccine hesitancy is a significant barrier as “even when a COVID-19 vaccine is available, it can be rejected for a variety of reasons.” Reluctance or refusal to vaccinate threatens progress in tackling vaccine-preventable diseases, and vaccine hesitancy is one of the world’s top-10 public health problems. Several factors may influence the acceptance or hesitancy of the COVID-19 vaccine [4]. Previous studies (conducted in the US, UK, Australia, Japan, Nigeria, and other countries) [1,5,6,7,8,9,10,11,12,13] have shown that reduced willingness to vaccinate is associated with females, lower socioeconomic status, unemployment, and less educated respondents. In addition, distrust of vaccines, concerns about unforeseen side effects in the future, and negative discussions about vaccines on social media may also cause people to hesitate about whether to get vaccinated [14,15].

**Figure 1:**
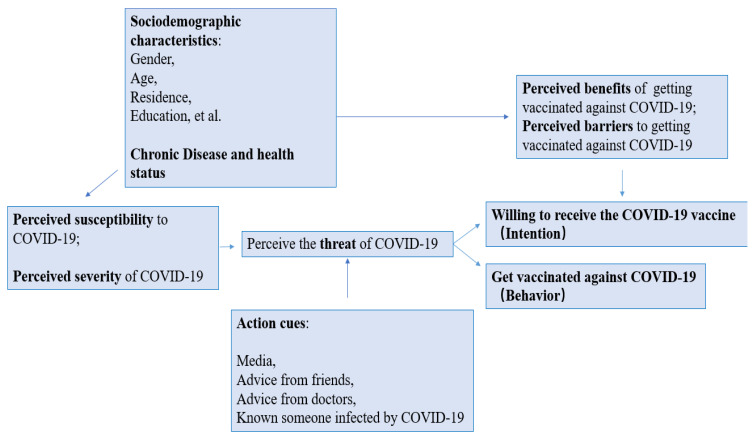
Conceptual framework of the determinants of COVID-19 vaccine acceptance (based on HBM).

The health belief model (HBM) is a conceptual framework widely used to study health beliefs that explain, predict, and influence behavior. HBM advises people to weigh the severity of the health threat they face (for example, perceived susceptibility and severity) against the perceived benefit or harm of taking a particular action related to that health threat (for example, vaccination) (Figure 1). Their risk assessment can be influenced by various factors, including action cues from trusted information sources and the social context in which they live and with which they interact. These factors have long been considered essential predictors of influenza vaccine uptake, and emerging studies suggest that they may also be necessary for COVID-19 vaccine uptake [16,17,18,19].

There is a real need for more research into the perceptions and acceptance of COVID-19 vaccines among Malawian residents, especially as the government is committed to a mass COVID-19 vaccination program. The purpose of this study was to investigate current vaccination rates for COVID-19 among Malawians, assess the level of COVID-19 vaccine hesitancy among Malawians, and explore the factors influencing vaccination and willingness to be vaccinated against COVID-19. The results of this study have important implications for the health sector when developing best practices for implementing COVID-19 vaccination programs, helping healthcare providers and policymakers to plan targeted education campaigns and vaccination awareness campaigns.

## 2. Materials and Methods

### 2.1. Study Design and Data

A cross-sectional design was used for this survey. The fieldwork was conducted in Lilongwe, Malawi, by the Peking University Research and Training Centre in Malawi (PKURTC) from 19 November to 30 November 2021. The target population were adults (aged 18 and above) living in Lilongwe, Malawi. Participants who had difficulties in communication and those who did not consent to the survey were excluded. A sample size of 693 was recommended, with an assumption of a 95% confidence interval (CI) regarding a 5% margin of error and a response rate of 60%. Participation was voluntary and came with no award, and all responses were anonymous. The final sample exceeded this estimate. A total of 758 questionnaires were collected and used for the analysis.

The study adopted a two-stage sampling technique consisting of the selection of residential areas and individuals. For the primary sampling unit, we used simple cluster sampling based on the list of Lilongwe’s administrative divisions (58 areas in total). As a result, 15 areas were selected from the list. Within each selected area, the sample sizes were population-weighted. We used systematic sampling of households according to house numbers and household heads in the survey.

A semi-structured questionnaire was used for the data collection. The questionnaire was deliberate, and some surveys regarding COVID-19 vaccination were conducted in other countries and reviewed by experts. It was initially prepared in English and then translated into Chichewa (see online Appendix A). The questionnaire was digitalized and programmed on tablets using Open Data Kit (ODK) software, version 1.28.4 (https://forum.getodk.org/ accessed on 11 April 2022). Investigators were assigned to each area and captured individual-level quantifiable indicators face to face.

The survey consisted of three sections: (1) general information and health status, including gender, age, education, residence, occupation, marital status, economic status, chronic disease, and history of vaccine rejection; (2) the health belief model, including two items on perceived susceptibility to COVID-19, two items on perceived severity, two items on the perceived benefits of getting vaccinated against COVID-19, one item on perceived barriers, and four items on action cues; (3) acceptance (intention and behavior) of the COVID-19 vaccine.

### 2.2. Measures

The dependent variable in this study was the acceptance of the COVID-19 vaccine, which was split into two parts: (1) behavior—taking the COVID-19 vaccine, and (2) intention—willing to get vaccinated, but has not yet received a vaccine. The rest were defined as vaccine unacceptance (had not taken or refused to take the COVID-19 vaccine). Therefore, the outcome variables were assessed with two items: “Have you taken a COVID-19 vaccine?” and “Would you accept or refuse a COVID-19 vaccine if it were offered to you?”.

We constructed independent variables based on the health belief model, including perceived susceptibility, perceived severity, perceived barriers, perceived benefits, action cues, and background factors (sociodemographic and disease history) of the HBM model. Each section consisted of several items, each item was answered yes/no, and each item was individually included in the regression analysis.

### 2.3. Statistical Analysis

Statistical analyses were performed in SPSS 25. Descriptive statistical analyses were used to characterize the study population. Correlation coefficients were calculated using χ2 to determine the association between the selected possible predictors and vaccination status or willingness to vaccinate. Those independent variables found to be statistically significant were included in the logistic regression model. A two-sided *p*-value of <0.05 was considered statistically significant. The final model was presented with adjusted odds ratios (OR), 95% confidence intervals (CI), and corresponding *p*-values.

Consent was sought from Lilongwe’s residents for participation before the questionnaire began. The study was designed and conducted according to the ethical principles established by Peking University. The National Committee on Research in the Social Sciences and Humanities, of The National Commission for Science and Technology, approved this study (P.08/21/593).

## 3. Results

A total of 758 people were included in the analysis, of which 189 (24.9%) were vaccinated, a further 271 (35.8%) were willing to be vaccinated but had not yet received the vaccine, and 298 (39.3%) refused to be vaccinated. The characteristics of the samples are shown in Table 1 and Table 2.

### 3.1. Sample Characteristics of Two Independent Classification Variables

#### 3.1.1. Demographic Characteristics

The study subjects comprised 498 (65.7%) females and 679 (89.6%) Christians. Most respondents were married (72.4%) and from rural areas (67.4%). One-third of the study participants were 25–34 years old. Among the respondents, 87.6% had a high school education level or below, while 11.9% had no education. Regarding their occupations, 38% had no job, while 3.6% of the respondents were healthcare workers. One-third of the study participants were in the lowest income category. In terms of health status, most of the population did not have any chronic diseases (79.4%), and only 2.9% considered themselves to be in poor health. A total of 4.5% of the participants reported having had COVID-19 before, while 21% had refused a vaccine recommended by a physician due to doubts.

As seen in Table 1, there were significant differences in COVID-19 vaccine acceptance among people of a different gender, age, education, occupation (health worker), monthly income, urban/rural residence, history of COVID-19 infection, and history of vaccine refusal. Table 2 also reflects a significant difference in COVID-19 vaccine acceptance among people with different attitudes toward the various components of the health belief model (perceived susceptibility, severity, benefits, barriers, and action cues).

#### 3.1.2. Health Benefit Model Characteristics

The majority of respondents agreed on the susceptibility, severity, and benefits of COVID-19 (more than 80%), with 86.8% agreeing that COVID-19 is contagious and 78.4% believing that they are likely to get it. About 92% of participants considered the consequences of COVID-19 to be serious, while 81.1% thought it would be beneficial to be vaccinated against COVID-19 to decrease the chance of contracting COVID-19 or suffering complications and in order to stop the spread of the virus in the community. A total of 76.3% perceived a barrier that prevented them from getting vaccinated. As for the action cues, 35.2% knew someone who had been infected. The majority (62.3%) heard information about vaccines from friends, and nearly half obtained information from the radio, while only 5.9% obtained it from healthcare providers. The results are shown in Table 2.

### 3.2. Influencing Factors Associated with the Acceptance of the COVID-19 Vaccine

The influencing factors for the acceptance of the COVID-19 vaccine are shown in columns 2–3 of Table 3. A Chi-square analysis of the sociodemographic and health-related variables revealed some significant variables. When entered into a binary logistic regression model, these variables were associated with “acceptance of COVID-19 vaccine”. In the final model, respondents aged 18–24 (OR = 5.079, 95% CI 2.303–11.202), 25–34 (OR = 2.723, 95% CI 1.363–5.438), urban residents (OR = 1.915, 95% CI 1.151–3.187), graduates/professionals (OR = 1.193, 95% CI 0.857–1.651), health workers (OR = 4.080, 95% CI 1.387–12.000), self-reporting health as good (OR = 4.08, 95% CI 1.410–11.840) and fair (OR = 3.145, 95% CI 1.063–9.308), perceived susceptibility (COVID-19 is contagious for you (OR = 1.787, 95% CI 1.226–2.605)), perceived benefit (agree that the vaccine could stop the spread of COVID-19 (OR = 2.992, 95% CI 1.851–4.834)), and action cues (know someone who has been infected by COVID-19 (OR = 2.001, 95% CI 1.285–3.115)) were predictors for the “acceptance of the COVID-19 vaccine”. Meanwhile, the historic rejection of vaccines (OR = 0.160, 95% CI 0.083–0.309) was an inhibitor of the “acceptance of the COVID-19 vaccine”.

### 3.3. Influencing Factors Associated with Positive Vaccination Intention and Behavior

According to the Chi-square calculation, it can be seen in Table 1 and Table 2 that positive vaccination intention and behavior are statistically correlated with gender, age, urban residents, education, employment, healthcare worker, monthly income, previous diagnosis of COVID-19, historic vaccine rejection, perceived susceptibility to COVID-19, perceived severity of COVID-19, perceived benefits and barrier to getting a COVID-19 vaccine, and action cues. Therefore, in a multinomial regression analysis, we only consider these significantly correlated variables as predictive variables.

As shown in columns 4–5 of Table 3, multinomial logistic regressions found that the promoters of vaccination behavior (Vaccinated) included age 18–24 (OR = 1.118, 95% CI 0.989–1.546), age 25–34 (OR = 1.391, 95% CI 0.853–1.684), urban residents (OR = 1.667, 95% CI 0.868–3.201), monthly income (0–50,000 MWK) (OR = 3.845, 95% CI 2.068–7.148), graduate/professional (OR = 4.343, 95% CI 0.940–20.044), healthcare worker (OR = 2.362, 95% CI 0.068–8.910), perceived susceptibility (COVID-19 is contagious for you) (OR = 1.285, 95% CI 1.147–1.554), perceived severity (OR = 9.959, 95% CI 1.049–95.575), perceived benefit (COVID-19 vaccine can stop the virus from spreading in communities and countries (OR = 2.876, 95% CI 1.057–7.829)), and action cues (know someone who has been infected by COVID-19 (OR = 2.022, 95% CI 1.174–3.480)).

According to columns 6–7 of Table 3, the promoters of vaccination intention (Willing to be vaccinated but not yet) included monthly income (0–50,000 MWK) (OR = 11.604, 95% CI 6.260–21.509), perceived susceptibility (COVID-19 is contagious for you) (OR = 2.532, 95% CI 1.423–4.505), and perceived benefit (COVID-19 vaccine can stop the virus from spreading in communities and countries (OR = 2.450, 95% CI 1.096–5.474)).

The rejection of a historic vaccine (OR = 0.12, v95% CI 0.057–0.250) (OR = 0.482, v95% CI 0.291–0.798) is an inhibitor of vaccination behavior and intention.

## 4. Discussion

This study explores the predictors of intention and behavior as they pertain to COVID-19 vaccines among adults in Lilongwe, Malawi, and the applicability of the health beliefs model. There are only previous studies about Malawian residents’ knowledge, attitudes, and practices regarding COVID-19 [3] and Malawian healthcare workers’ vaccination status [20].

This study shows that perceived susceptibility and perceived benefit in the HB8M model are essential factors for promoting COVID-19 vaccine acceptance, improving people’s vaccination intention, and promoting people’s vaccination behavior. Perceived severity and crucial action cues such as knowing someone who has had COVID-19 can improve vaccination acceptance by promoting vaccination behavior. Perceived impairment did not play a role in this study. Consistent with previous research [21,22,23], the main dimensions of the HBM model were almost all related to COVID-19 vaccine acceptance. However, our study distinguished between the different facilitation effects of different dimensions on vaccination intention and behavior.

In addition, as background factors that may be involved in vaccination decision making in the HBM model, we also analyzed their potential influence on vaccination intention and behavior. In the current study, those aged between 18 and 34, graduates/professionals, and healthcare workers had more active vaccination behavior. The high acceptance of the COVID-19 vaccine among healthcare workers is consistent with another study on COVID-19 vaccination among healthcare workers in Malawi [20]. Likewise, other studies have found that young people and those with higher education levels are more likely to be vaccinated [24,25]. We presume that this is possibly because they were given more information about vaccines and were better able to make informed decisions. In addition, people with lower monthly incomes have a higher acceptance of the COVID-19 vaccine, which is consistent with some previous studies [26,27,28]. This is widely believed to be due to the government’s policy of free vaccines.

According to the results of this study, the most widely available sources of information about COVID-19 vaccines are the radio and friends. There is little information from doctors and a lot of ignorance or incorrect knowledge about vaccines, which has led to distrust and the rejection of COVID-19 vaccines among Malawians [29]. Therefore, Malawi should be supported in its vaccination outreach and community mobilization campaigns to raise awareness of COVID-19 through radio programs, jingles, and volunteer door-to-door outreach services [30]. The education of the population should be strengthened regarding their vulnerability to COVID-19 infection. People need to be aware of existing health risks, feel at risk, and take protective measures. The benefits of vaccination also need to be highlighted. People need to be aware that vaccines protect them and their communities. Additionally, we can spread information on real-life COVID-19 cases and successful vaccination stories to promote vaccination behavior. We should also track and address rumors/misinformation about COVID-19 vaccines to rebuild public confidence in vaccination. At the same time, Malawi has its own unique cultural and religious background, so it is essential to work with trusted community leaders. Religious leaders can also act as vaccine advocates, using existing trust relationships to advocate for vaccination [31,32].

Urban residents have more active vaccination behavior because it is more challenging to get vaccines for people who live in rural areas compared with urban areas. Thus, Malawi needs to improve access to vaccines for rural residents. We suggest targeted improvements in infrastructure, including logistics for vaccine transport and distribution [33,34], such as “MetaFridge”, a portable ice tub for cryostorage and delivery. The preponderance of convenient vaccination sites should also be increased, especially in rural areas. International organizations and local governments should work together to cover the “last mile” of vaccination. This will also facilitate the establishment of long-term interventions and adaptive infrastructure that can be used for future disease control efforts.

We found that there are still gaps between COVID-19 vaccination intention and behavior. This suggests that real-world conditions may limit vaccination opportunities or that willing individuals may hesitate when vaccines become available. These issues should be addressed when planning vaccination campaigns. Last year, the Malawi government developed a new plan called the National COVID-19 Strategy and Plan—July 2021–June 2022 [35], which builds on the successes achieved and lessons learned from previous plans. The plan includes future control strategies for inter-cluster coordination, health, education, public communication, local governance, protection and social support, employment and labor force protection, transport and logistics, and security and enforcement. It focuses on moving from emergency to longer-term interventions and building from semi-permanent to permanent adaptive infrastructure. Our findings are consistent with ongoing strategies, particularly government-led advocacy, education, and infrastructure development.

This study has several limitations. Firstly, the results of this study may not represent the views or practices of the population as a whole. Secondly, given the cross-sectional nature of the data, the results represent a snapshot of vaccine indecision at one point in time. We cannot explain how attitudes will evolve as the COVID-19 pandemic, vaccine availability, and political discourse change. Thirdly, there is an underlying social desirability bias, according to which participants may react in ways that they think are acceptable. Additionally, we did not assess the impact of rapid mutations of SARS-CoV-2 on COVID-19 vaccine uptake. For example, new mutant strains such as Delta and Omicron may re-infect people who have already been vaccinated with previous vaccines, which may negatively affect people’s views on vaccination [36].

## 5. Conclusions

Overall, vaccine acceptance (including those who have been vaccinated and those who are willing to be vaccinated) was not high enough among the respondents to protect themselves and their communities. The health belief model structure can be used as a good predictor of vaccine acceptance, especially “perceived susceptibility”, “perceived benefit”, and “action cues”. Strengthening COVID-19 vaccine education in these areas will be an essential future intervention.

## Figures and Tables

**Table 1:** Demographic characteristics and *p*-values of the samples.

Variables	Total N = 758		Vaccine Acceptance N = 460				Vaccine Unacceptance N = 189		*p*-Value
			Vaccinated		Willing to be vaccinated but not yet been vaccinated				
			N = 189		N = 271				
	*n*	%	*n*	%	*n*	%	*n*	%	
Sociodemographic characteristics									
Gender									0.012 *
Male	260	34.3	80	30.8	93	35.8	87	33.5	
Female	498	65.7	109	21.9	178	35.7	211	42.4	
Age									<0.001 *
18–24	173	22.8	23	13.3	63	36.4	87	50.3	
25–34	263	34.7	62	23.6	101	38.4	100	38	
35–44	162	21.4	55	34	49	30.2	58	35.8	
45–54	80	10.6	21	26.3	31	38.8	28	35	
55 and above	80	10.6	28	35	27	33.8	25	31.3	
Residence									<0.001 *
Urban	246	32.5	98	39.8	66	26.8	82	33.3	
Rural	512	67.5	91	17.8	205	40	216	42.2	
Religion									0.275
Christian	679	89.6	174	25.6	240	35.3	265	39	
Islam	35	4.6	10	28.6	11	31.4	14	40	
Other(African traditional religion/Chewa/None)	44	5.8	5	11.4	20	45.5	19	43.2	
Marital status									0.089
Married	549	72.4	126	23	191	34.8	232	42.3	
Never married	114	15	32	28.1	41	36	41	36	
Divorced	58	7.7	17	29.3	25	43.1	16	27.6	
Widowed	37	4.9	14	37.8	14	37.8	9	24.3	
Education									<0.001 *
No high school	360	47.5	59	16.4	150	41.7	151	41.9	
High school	214	28.2	58	27.1	68	31.8	88	41.1	
College	64	8.4	31	48.4	16	25	17	26.6	
Graduate/Professional	30	4	21	70	3	10	6	20	
Not educated	90	11.9	20	22.2	34	37.8	36	40	
Employment									<0.001 *
Government employee	27	3.6	20	74.1	1	3.7	6	22.2	
Nongovernment employee	70	9.2	26	37.1	22	31.4	22	31.4	
Self-employed	197	26	56	28.4	61	31	80	40.6	
Student	18	2.4	5	27.8	9	50	4	22.2	
Retired	7	0.9	5	71.4	1	14.3	1	14.3	
Unemployed	288	38	51	17.7	102	35.4	135	46.9	
Other	151	19.9	26	17.2	75	49.7	50	33.1	
Healthcare worker									<0.001 *
Yes	27	3.6	21	77.8	2	7.4	4	14.8	
No	731	96.4	168	23	269	36.8	294	40.2	
Monthly income(MWK)									<0.001 *
0–25,000	502	66.2	144	28.7	230	45.8	128	25.5	
25,000–50,000	98	12.9	21	21.4	26	26.5	51	52	
50,000 and above	158	20.8	24	15.2	15	9.5	119	39.3	
Health characteristics									
Chronic disease									0.380
Yes	156	20.6	44	28.2	49	31.4	63	40.4	
No	602	79.4	145	24.1	222	36.9	235	39	
Self-reported health									0.065
Good	535	70.6	129	24.1	205	38.3	201	37.6	
Fair	201	26.5	51	25.4	59	29.4	91	45.3	
Poor	22	2.9	9	40.9	7	31.8	6	27.3	
Ever diagnosed with COVID-19									0.015 *
Yes	34	4.5	15	44.1	12	35.3	7	20.6	
No	724	95.5	174	24	259	35.8	291	40.2	
Historic vaccine rejection									<0.001 *
Yes	159	21	13	8.2	43	27	103	64.8	
No	599	79	176	29.4	228	38.1	195	32.6	

* *p* < 0.05.

**Table 2:** Health benefit model characteristics and *p*-values of the samples.

Variables	Total N = 758		Vaccine Acceptance N = 460				Vaccine Unacceptance N = 189		*p*-Value
			Vaccinated		Willing to be vaccinated but not yet been vaccinated		87		
			N = 189		N = 271				
	n	%	n	%	n	%	n	%	
Perceived susceptibility to COVID-19									
Do you agree that COVID-19 is contagious?									<0.001 *
Agree	658	86.8	181	27.5	248	37.7	229	34.8	
Disagree	100	13.2	8	8	23	23	69	69	
Do you think getting COVID-19 is currently a possibility for you?									<0.001 *
Agree	594	78.4	157	26.4	240	40.4	197	33.2	
Disagree	164	21.6	32	19.5	31	18.9	101	61.6	
Perceived severity of COVID-19									
Do you agree that the COVID-19 pandemic poses a risk to people in Malawi?									<0.001 *
Agree	698	92.1	186	26.6	259	37.1	253	36.2	
Disagree	60	7.9	3	5	12	20	45	75	
Do you agree that the consequences of getting COVID-19 can be serious and could even lead to death?									<0.001 *
Agree	697	92	188	27	257	36.9	252	36.2	
Disagree	61	8	1	1.6	14	23	46	75.4	
Perceived benefits of getting vaccinated against COVID-19									
Do you agree that a COVID-19 vaccine can decrease your chances of contracting COVID-19 or suffering from complications?									<0.001 *
Agree	615	81.1	177	28.8	239	38.9	199	32.4	
Disagree	143	18.9	12	8.4	32	22.4	99	69.2	
Do you agree that a COVID-19 vaccine can stop the virus from spreading within communities and between countries?									<0.001 *
Agree	615	81.1	178	28.9	233	37.9	204	33.2	
Disagree	143	18.9	11	7.7	38	26.6	94	65.7	
Perceived barriers to getting vaccinated against COVID-19									
Do you agree that immunization requirements go against freedom of choice?									0.064
Agree	578	76.3	146	25.3	194	33.6	238	41.2	
Disagree	180	23.7	43	23.9	77	42.8	60	33.3	
Action cues									
Do you know someone who has been infected by COVID-19?									<0.001 *
Yes	267	35.2	107	40.1	75	28.1	85	31.8	
No	491	64.8	82	16.7	196	39.9	213	43.4	
Have you received information about COVID-19 and vaccines from friends?									0.001 *
Yes	472	62.3	133	28.2	149	31.6	190	40.3	
No	286	37.7	56	19.6	122	42.7	108	37.8	
Have you received information about COVID-19 and vaccines from healthcare providers?									0.791
Yes	45	5.9	14	31.1	17	37.8	14	31.1	
No	713	94.1	175	24.5	254	35.6	284	39.8	
Have you received information about COVID-19 and vaccines from the radio?									0.042 *
Yes	385	50.8	106	27.5	123	31.9	156	40.5	
No	373	49.2	83	22.3	148	39.7	142	38.1	

* *p* < 0.05.

**Table 3:** Outcomes of logistic regression (ref: Vaccine unacceptance).

Variables	Binary Logistic Regression		Multinomial Logistic Regression			
	Acceptance of COVID-19 Vaccine		Vaccinated		Willing to Be Vaccinated but Not Yet Been Vaccinated	
	aOR (95% CI)	*p*-Value	OR (95% CI)	*p*-Value	OR (95% CI)	*p*-Value
Demographic characteristics						
Age						
18–24	5.079 (2.303–11.202)	<0.001 *	1.181 (0.989–1.546)	0.001 *	1.46 (0.621–1.725)	0.386
25–34	2.723 (1.363–5.438)	0.005 *	1.391 (0.835–1.684)	0.044 *	0.898 (0.396–2.038)	0.798
35–44	1.057 (0.537–2.079)	0.872	0.83 (0.372–1.851)	0.65	1.058 (0.450–2.487)	0.898
45–54	1.802 (0.815–3.985)	0.146	0.584 (0.237–1.440)	0.243	0.924 (0.355–2.406)	0.872
55 and above	1		1		1	
Residence						
Urban	1.915 (1.151–3.187)	0.012 *	1.667 (0.868–3.201)	0.025 *	0.626 (0.341–1.149)	0.131
Rural	1		1		1	
Education						
No high school	1.634 (0.849–3.137)	0.141	0.669 (0.302–1.483)	0.322	0.959 (0.491–1.873)	0.902
High school	0.994 (0.475–2.080)	0.986	0.972 (0.397–2.376)	0.950	1.25 (0.571–2.733)	0.577
College	0.664 (0.254–1.733)	0.403	1.508 (0.442–5.057)	0.519	0.948 (0.300–2.996)	0.928
Graduate/Professional	1.193 (0.857–1.651)	0.008 *	4.342 (0.940–20.044)	0.040 *	1.82 (0.317–10.644)	0.502
Not educated	1		1		1	
Healthcare worker						
Yes	4.080 (1.387–12.000)	0.011 *	2.362 (0.602–8.910)	0.002 *	0.237 (0.034–1.646)	0.133
No	1		1		1	
Monthly income (MWK)						
0–50,000	1.982 (0.991–4.030)	0.060	3.845 (2.068–7.148)	<0.000 *	11.604 (6.260–21.509)	<0.000 *
50,000 and above	1		1		1	
Health status and vaccine history						
Self-reported health						
Good	4.08 (1.410–11.840)	0.01 *	0.394 (0.098–1.577)	0.188	1.475 (0.377–5.677)	0.576
Fair	3.145 (1.063–9.308)	0.038 *	0.326 (0.081–1.320)	0.116	0.738 (0.186–2.925)	0.665
Poor	1		1		1	
Historic vaccine rejection						
Yes	0.160 (0.083–0.309)	<0.001 *	0.120 (0.057–0.250)	<0.000 *	0.482 (0.291–0.798)	0.005 *
No	1		1		1	
HBM characteristics						
Perceived susceptibility						
COVID-19 is contagious for you						
Agree	1.787 (1.226–2.605)	0.003 *	2.012 (0.772–5.244)	0.013 *	2.532 (1.423–4.505)	0.002 *
Disagree	1		1		1	
			Perceived severity			
COVID-19 can be serious and can even lead to death						
Agree	2.137 (0.904–4.113)	0.087	9.959 (1.049–95.575)	0.045 *	0.925 (0.370–2.316)	0.868
Disagree	1		1		1	
Perceived benefits						
A COVID-19 vaccine can stop the virus from spreading within communities and between countries						
Agree	2.992 (1.851–4.834)	<0.001 *	2.876 (1.057–7.829)	0.039 *	2.450 (1.096–5.474)	0.029 *
Disagree	1		1		1	
Action cues						
Known someone infected by COVID-19						
Yes	2.001 (1.285–3.115)	0.002 *	2.022 (1.174–3.480)	0.011 *	0.965 (0.587–1.584)	0.887
No	1		1		1	

Abbreviations: OR = odds ratio; aOR = adjusted odds ratio; CI = confidence interval. * *p*-values < 0.05 were considered statistically significant.

## Data Availability

All data generated during this study are included in this published article and Appendix A.

## Appendix A. Data Collection Tools

English Questionnaire.

**Table A1:** COVID-19 vaccination acceptance questionnaire.

Interviewer ID: ________		Questionnaire ID: ________	Date: _______________
**Section A: General Information and Health Status**			
1. Gender		1.1	Male
		1.2	Female
2. Year of Birth		2.1	18–24
		2.2	25–34
		2.3	35–44
		2.4	45–54
		2.5	55 and above
3. Residence		3.1	Urban
		3.2	Rural
Indicate area of residence			
4. Religion		4.1	Christian
		4.2	Islam
		4.3	Other
Other			
5. Marital status		5.1	Married
		5.2	Never married
		5.3	Divorced
		5.4	Widowed
6. Education Attained		6.1	No high school
		6.2	High school
		6.3	College
		6.4	Graduate/Professional
		6.5	Not educated
7. Employment Status		7.1	Government employee
		7.2	Nongovernment employee
		7.3	Self-employed
		7.4	Student
		7.5	Retired
		7.6	Unemployed
		7.7	Other (specify)
8. Are you a healthcare worker?		8.1	Yes
			(If Yes)
		8.11	Physician,
		8.12	Nurse,
		8.13	Health Official,
		8.14	Researcher,
		8.15	Other
		8.2	No
9. What category (MWK) best fits your overall monthly income?		9.1	0–5000
		9.2	5000–10,000
		9.3	10,000–25,000
		9.4	25,000–50,000
		9.5	50,000 and above
10. How many people live in your household (HH member should have lived at household for at least six months or more)?		10.1	1–3
		10.2	4–5
		10.3	5 and above
11. How would you describe your house’s condition? **[check multiple answer option]**		11.1	We have electricity, and it functions at least half a day per day.
		11.2	We have a safe, clean water source (piped into dwelling or borehole with pump or protected dug well).
		11.3	We have toilets in good condition (flush or ventilated improved latrine).
		11.4	We are not crowded (5 or fewer people per room).
		11.5	We have a firm roo f(tiles or galvanized iron or concrete).
		11.6	Nothing
12. What does the household own as a family? **[check multiple answer option]**		12.1	Radio
		12.2	Television
		12.3	Stove
		12.4	Fridge
		12.5	Sofa
		12.6	Mobile phone
		12.7	Bicycle
		12.8	Car
		12.9	Motorbike
		12.10	oxcart
		12.11	Small livestock, e.g., poultry, goats, pigs
		12.12	Large livestock: cattle
		12.13	None
		12.14	other(specify)
13. Do you suffer from any chronic diseases?		13.1	Yes
		13.2	No
14. How would you perceive your overall health?		14.1	Good
		14.2	Fair
		14.3	Poor
15. Have you been (or are you currently) infected with COVID-19?		15.1	Yes
		15.2	No
16. Do you personally know someone who has been (or is currently) infected with COVID-19?		16.1	Yes
		16.2	No
17. Have you ever refused a vaccine recommended by a physician due to doubts you had about it?		17.1	Yes
		17.2	No
		17.3	Have never heard of any vaccine
**Section 2: Perceived susceptibility, severity and benefits of COVID-19 vaccine**			
18.Have you ever heard about a COVID-19 vaccine?		18.1	Yes
		18.2	No
19. Where do you obtain COVID-19 and vaccine information? **[check multiple answer option]**		19.1	Internet/TV
		19.2	Visits to healthcare providers
		19.3	Family members
		19.4	Radio
		19.5	School
		19.6	Church
		19.7	Work place
		19.8	Friends
		19.9	Printed materials from healthcare providers
		19.10	Vaccine companies and industry
		19.11	Others(specify)
20. To what extent do you agree that COVID-19 is contagious?		20.1	Strongly agree
		20.2	Agree
		20.3	Disagree
		20.4	Strongly disagree
21. To what extent do you agree that the COVID-19 pandemic poses a risk to people in Malawi?		21.1	Strongly agree
		21.2	Agree
		21.3	Disagree
		21.4	Strongly disagree
22. Do you agree that the consequences of getting COVID-19 can be serious and can even lead to death?		22.1	Strongly agree
		22.2	Agree
		22.3	Disagree
		22.4	Strongly disagree
23. Do you think getting COVID-19 is currently a possibility for you?		23.1	Strongly agree
		23.2	Agree
		23.3	Disagree
		23.4	Strongly disagree
24. Do you agree that a COVID-19 vaccine can decrease the chances of you contracting COVID-19 or suffering from its complications?		24.1	Strongly agree
		24.2	Agree
		24.3	Disagree
		24.4	Strongly disagree
25. Do you agree that a COVID-19 vaccine can stop the virus from spreading within communities and between countries?		25.1	Strongly agree
		25.2	Agree
		25.3	Disagree
		25.4	Strongly disagree
26. Do you agree that a COVID-19 vaccine should be compulsory for all citizens and residents in Malawi?		26.1	Strongly agree
		26.2	Agree
		26.3	Disagree
		26.4	Strongly disagree
27. Do you agree that immunization requirements go against freedom of choice?		27.1	Strongly agree
		27.2	Agree
		27.3	Disagree
		27.4	Strongly disagree
**Section 3: Acceptability and Practice of a COVID-19 Vaccine**			
28.a Have you been vaccinated with a COVID-19 vaccine?			Yes
			No
28.b Why did you choose to get vaccinated?		1–10	I was forced to do it.
			I want to travel outside.
			I got sick.
			Someone I knew died of COVID-19.
			Requirement for me to start job.
			Peer pressure.
			I was convinced by medical personnel.
			I want to protect myself from COVID
			I want to protect my family from COVID
			Other()
28.c Would you accept or refuse a COVID-19 vaccine when if it were available?		28.1	I would **accept immediately**. [Go to Q31]
		28.2	I would **delay** the vaccination. [Go to Q29]
		28.3	I would **refuse** the vaccination. [Go to Q29]
29. I would delay or refuse the vaccination because-**[check multiple answer option]**		29.1	I don’t believe in the existence of COVID-19.
		29.2	I think the vaccine is a plot.
		29.3	I am religious and God will protect me.
		29.4	COVID-19 symptoms are mostly mild so I do not fear COVID-19.
		29.5	I feel that masks and sanitizers are sufficient for protection.
		29.6	I think the vaccine will transmit the virus to me.
		29.7	I think the vaccine will change my genes.
		29.8	I don’t think that I can afford the vaccine.
		29.9	The fear of adverse side effects.
		29.10	Not convinced that it will be effective.
		29.11	Concern regarding the faulty/fake COVID-19 vaccines.
		29.12	The speed of developing the vaccine was too fast.
		29.13	The short duration of clinical trials.
		29.14	There is no way I trust governments.
		29.15	Illness or allergy prevented me from getting vaccinated.
		16	I feel the COVID vaccine is associated with other religious hidden agendas.
		17	Others()
30. I would take the COVID-19 vaccine only if-**[check multiple answer option]**		30.1	I were given adequate information about it.
		30.2	The vaccine were taken by many people.
		30.3	The vaccine’s safety were confirmed.
		30.4	The vaccine were provided for free.
		30.5	The doctor advised me to get vaccinated.
		30.6	The government required me to get vaccinated.
		30.7	The WHO or UNICEF staff provided me with a vaccine.
		30.8	No vaccinations at all.
		30.9	Other
31 How much are you willing to pay for COVID-19 vaccines?		MWK	
32. How much do you know about and trust the following vaccines?			
Manufacture	I don’t know at all	I’ve heard of it but I don’t trust it	I know about it and I trust it
AstraZeneca/Oxford vaccine [UK]			
Johnson and Johnson [US]			
Moderna [US]			
NOVAVAX [US]			
Janssen [US]			
Pfizer/BionTech [China]			
Sinovac [China]			
IMBCAMS [China]			
Zhifei Longcom [China]			
Sinopharm Beijing [China]			
CanSinoBIO [China]			
THE GA Vector State Research Centre of Viralogy and Biotechnology [Russia]			
MALEYA NATIONAL CENTER [Russia]			
Serum Institute [India]			

**Chichewa Translated COVID-19 Vaccination Acceptance Questionnaire.**

**Mafunso wokhudza Kuvomereza Katemera wa COVID-19.**

**Malonje.**

Dzina langa ndine____________________ ndachokela ku ____________ amene tikupanga zakafukufuku pa zomwe mumadziwa pankhani ya Katemera wa COVID-19. Zokambilana zathu zikhala muzigawo ziwiri zotele: gawo la Zokhudza muthu ndi umoyo wake; ndi gawo la Maganizo pakukhudziwa, Muyenso komanso kufunika kwa Katemera wa COVID-19.

Macheza athu atenga pafupifupi mpindi nkhumi ndi zisanu ndipo zonse zimene tikhale tikukambilana zikhala zachinsinsi. Kutengapo mbali kwanu mukafukufuku ameneyu ndikosakakamiza ndipo mukhoza kukana kutenga nawo mbali kapena kusiila panjira macheza amenewa. Ngati mwasankha kuti simutenga nawo mbali pakafukufuku ameneyu simulandila chilango chilichonse kapena kulandidwa Katundu aliyense. Chizindikiilo chanu cha mzika chingowilitsidwa ntchito pakungoonetsa kuti inuyo munavomeleza kuchita nawo kafukufuku ameneyu, koma sichidzagwilitsadwanso ntchito penapaliponse.

Mukuvomeleza kutenga nawo mbali pakafukufuku ameneyi?

Inde Ayi.

**Table A2:** Mafunso wokhudza Kuvomereza Katemera wa COVID-19. Malonje.

ID ya Ofunsa Mafunso: ________		Questionnaire ID: ________	Tsiku: _______________
**Gawo 1: Zokhudza Muthu Ndi Umoyo Wake**			
1. Mamuna/Mkazi		1.1	Mamuna
		1.2	Mkazi
2. Muli ndi zaka zingati?		2.1	18–24
		2.2	25–34
		2.3	35–44
		2.4	45–54
		2.5	55 kupita mtsogolo
3. Dela lokhala		3.1	Mtawuni
		3.2	Kumudzi
4. Ndinu achipembedzo chanji?		4.1	Chikhilisitu
		4.2	Chisilamu
		4.3	Zina (Tchulani)
5. Muli pa banja?		5.1	Ndili pa banja
		5.2	Sinnakwatirepo
		5.3	Banja linatha
		5.4	Wa masiye
6. Maphuzilo anu munalekezera pati?		6.1	Sanafike sekondale
		6.2	Sekondale
		6.3	Kunapita ku Koleji
		6.4	Anaphunzira ku university
7. Mumagwira ntchito yanji?		7.1	Amagwira ntchito mu boma
		7.2	Amagwira ntchito kumabugwe
		7.3	Anazilemba okha ntchito
		7.4	Mwana wasukulu
		7.5	Anapanga litaya
		7.6	Sali pa ntchito
		7.7	Zina (Tchulani)
8. Kodi ndinu ogwira ntchito za umoyo (zachipatala)?		8.1	Eya
			**(ngati eya)**
		8.11	Owona odwala (Physicians),
		8.12	Nurses,
		8.13	Oyang’anira za umoyo (Health Officials),
		8.14	Opanga zakafukufuku (Researchers),
		8.15	Zina (Others)
		8.2	Ayi
9. Mumapeza ndalama zingati pa mwezi??		9.1	0–5000
		9.2	5000–10,000
		9.3	10,000–25,000
		9.4	25,000–50,000
		9.5	50,000 kupita mtsogolo
10. Pakhomo pano pamakhala anthu angati (Okhala pakhomo akhale amene wakhala pabanjapo posachepera miyezi isanu ndi umodzi)?		10.1	1–3
		10.2	4–5
		10.3	5 kupita mtsogolo
11. Kodi nyumba yanu mungayifotokoze bwanji? **[Mayankho ambiri ndi ololedwa]**		11.1	Tili ndi magetsi, atha kukhala akuyaka mafupifupi theka la tsiku, tsiku lililonse.
		11.2	Tili ndi kochokera madzi awukhondo (Madzi a muma paipi, Mijigo kapena zitsime zotetezedwa)
		11.3	Tili ndi zimbudzi za ukhondo komanso zabwino (Zo flasha komanso zopita mphweya).
		11.4	Sitili odzadzana (Timakhala athu ochepera 5 (asanu) mu chipinda).
		11.5	Tili ndi denga lolimba (tiles or malata kapena concrete).
12. Kodi Pakhomo pano, muli ndi zinthu ziti ngati banja? **[Mayankho ambiri ndi ololedwa]**		12.1	Radio
		12.2	Television
		12.3	Stove
		12.4	Fridge
		12.5	Sofa
		12.6	Mobile phone
		12.7	Njinga
		12.8	Galimoto
		12.9	Njinga yamoto
		12.10	Ngolo
		12.11	Zina (Tchulani)
13. Mumadwala matenda a mgonagona?		13.1	Yes
		13.2	Ayi
14. Mukuona ngati umoyo wanu uli bwanji?		14.1	Uli bwino
		14.2	Uli pakatikati
		14.3	Suli bwino
15. Kodi munapezekako kapena padali pano muli ndi COVID-19?		15.1	Eya
		15.2	Ayi
16. Mukudziwako wina wake amene anali kapena ali ndi COVID-19, mbanja mwanu kapena mdera lanu lino?		16.1	Eya
		16.2	Ayi
17. Kodi munakanako katemera chifukwa chakumukayikira?		17.1	Eya
		17.2	Ayi
**Gawo 2: Maganizo pakukhudziwa, Muyenso komanso kufunika kwa Katemera wa COVID-19**			
18. Munamvako zaketemera wa COVID-19?	18.1	Eya	
	18.2	Ayi	
19. Kodi mauthenga okhudza COVID-19 ndi katemera mungaupeze kuti? **[Mayankho ambiri ndi ololedwa]**	19.1	Intaneti/TV	
	19.2	Kupita Kumalo othandizira odwala	
	19.3	Akubanja ndi azibale	
	19.4	Azanga	
	19.5	Zolemba kapena ma poster opangidwa ndi achipatala	
	19.6	Ma Kampani opanga katemera	
	19.7	Wayilesi	
	19.8	Ku sukulu	
	19.9	Kuntchito	
	19.91	Tchalitchi	
	19.92	Zina (Tchulani)	
20. Kodi mukugwirizana nazo bwanji kuti matenda a COVID-19 ndiopatsirana?	20.1	Ndikugwilizana nazo kwambiri	
	20.2	Ndikugwilizana nazo	
	20.3	Sindikugwilizana nazo	
	20.4	Sindikugwilizana nazo konse	
21. Kodi mukugwirizana nazo bwanji kuti matenda a COVID 19 ayika pachiopsezo anthu (mtundu wa) aku Malawi?	21.1	Ndikugwilizana nazo kwambiri	
	21.2	Ndikugwilizana nazo	
	21.3	Sindikugwilizana nazo	
	21.4	Sindikugwilizana nazo konse	
22. Mukugwirizana nazo bwanji kuti zotsatira za matenda a COVID-19 atha kukhala oopsa mpaka munthu kumwalira?	22.1	Ndikugwilizana nazo kwambiri	
	22.2	Ndikugwilizana nazo	
	22.3	Sindikugwilizana nazo	
	22.4	Sindikugwilizana nazo konse	
23. Mukuona ngati padali pano, ndizotheka kutenga matenda a COVID-19?	23.1	Ndikugwilizana nazo kwambiri	
	23.2	Ndikugwilizana nazo	
	23.3	Sindikugwilizana nazo	
	23.4	Sindikugwilizana nazo konse	
24. Mukugwirizana zano bwanji kuti katemera wa COVID 19 atha kuchepetsa kuthekera kotenga matenda a COVID-19 kapena zotsatira zake?	24.1	Ndikugwilizana nazo kwambiri	
	24.2	Ndikugwilizana nazo	
	24.3	Sindikugwilizana nazo	
	24.4	Sindikugwilizana nazo konse	
25. Mukugwirizana nazo bwanji kuti katemera wa COVID-19 atha kuletsa (kusiyiza) kufalikira kwa matendawa mu mmadera amayiko?	25.1	Ndikugwilizana nazo kwambiri	
	25.2	Ndikugwilizana nazo	
	25.3	Sindikugwilizana nazo	
	25.4	Sindikugwilizana nazo konse	
26. Kodi mukugwirizana nazo bwanji kuti Katemera wa COVID-19 azikhala wokakamiza kwa nzika zonse ndi okhala mdziko muno?	26.1	Ndikugwilizana nazo kwambiri	
	26.2	Ndikugwilizana nazo	
	26.3	Sindikugwilizana nazo	
	26.4	Sindikugwilizana nazo konse	
27. Mukugwirizana nazo bwanji ndizakuti zofunika poziteteza zimatsutsana ndi ufulu wachisankho?	27.1	Ndikugwilizana nazo kwambiri	
	27.2	Ndikugwilizana nazo	
	27.3	Sindikugwilizana nazo	
	27.4	Sindikugwilizana nazo konse	
**Gawo 3: Kuvomereza komanso zochita kukhudza katemera wa COVID-19**			
28. Kodi mutha kulola kapena kukana katemera wa COVID-19, atapezeka ?	28.1	Nditha kulora pompo pompo. [pitan ku Q31]	
	28.2	Nditha kuchedwa kuvomera [pitan ku Q29]	
	28.3	Nditha kukana katemerayi. [pitan ku Q29]	
29. Nditha kuchedwa kuvomera ka kukana katemerayi, chifukwa: **[Mayankho ambiri ndi ololedwa]**	29.1	Sindikhulupilira kutI COVID-19 ilipo.	
	29.2	I think the vaccine is a plot.	
	29.3	Ndine wachipembedzo, mulungu anditeteza.	
	29.4	Zizindikiro za matenda a COVID-19 amakhala osaopsa kwenikweni, sindiopa za COVID-19.	
	29.5	Ndimaona ngati masiki ndi hand sanitizer ndizokwanira kunditeteza ku matendawa.	
	29.6	Ndikuona ngati katemera atha kundipatsira matenda a COVID-19.	
	29.7	Ndikuona ngati katemera atha kusintha ma genes anga	
	29.8	Sindingakwanitse kulipira katemerayu.	
	29.9	Ndimaopa zotsatira zoopsa za katemerayi.	
	29.10	Sindine okhutitsidwa kuti katemerayu atha kugwira ntchito	
	29.11	Ndimaopa kuti katemera wina atha kukhala wa fake.	
	29.12	Ndikuona ngati katemerayu wapangidwa mwachangu.	
	29.13	Katemerayu sanayezedwe mokwanira.	
	29.14	Boma sindilikhulupilira.	
	29.15	Matenda kapena zowenga zinandiletsa kusabayitsa katemerayi.	
30. Nditha kukabayitsa katemera wa COVID-19, pokhapokha: **[Mayankho ambiri ndi ololedwa]**	30.1	Ndapatsidwa uphungu ndi uthenga okwanira.	
	30.2	Katemera akubayitsidwa ndi athu ambiri.	
	30.3	Chitetezo cha katemerayi ndichosakayikitsa/chostimikizidwa.	
	30.4	Katemera atakhala waulere.	
	30.5	Adotolo andilangiza kukabayitsa katemera.	
	30.6	Malamulo a bowa akundiyenera kuti ndibayitse katemera.	
	30.7	Mabugwe a WHO kapena UNICEF ndiamene akubaya katemerayu.	
	30.8	Palibe katemerayu ndikomwe.	
	30.9	Zina (Tchulani)	
31. Mutha kulipira ndalama zingati, kutakhala kuti katemera wa COVID-19 yu ndiwolipilitsa?			
		**MK**____________________________________	
32. Mumadziwa bwanji ndi kukhulupilira mitundu yamakatemera awa?			
Opanga	Sindidziwa kathu	Nnamvako koma sindimukhulupilira	Ndikumudziwa komanso ndimamukhulupirira
AstraZeneca/Oxford vaccine [UK]			
Johnson and Johnson [US]			
Moderna [US]			
NOVAVAX [US]			
Janssen [US]			
Pfizer/BionTech [China]			
Sinovac [China]			
IMBCAMS [China]			
Zhifei Longcom, [China]			
Sinopharm Beijing [China]			
CanSinoBIO [China]			
THE GA Vector State Research Centre of Viralogy and Biotechnology [Russia]			
MALEYA NATIONAL CENTER [Russia]			
Serum Institute [India]			
